# Population genetics of *Babesia vogeli* based on the mitochondrial cytochrome b gene

**DOI:** 10.1038/s41598-024-72572-z

**Published:** 2024-09-20

**Authors:** Ansu Kumari, Divya Agnihotri, Anil Kumar Nehra, Aman Dev Moudgil, Yudhbir Singh, Devendra Prasad Pateer, Rajat Garg

**Affiliations:** 1 Department of Veterinary Medicine, Lala Lajpat Rai University of Veterinary and Animal Sciences, Hisar, 125004 Haryana India; 2 Department of Veterinary Medicine, College of Veterinary Science, Guru Angad Dev Veterinary and Animal Sciences University, Rampura Phul, 151103 Punjab India; 3 Department of Veterinary Parasitology, Lala Lajpat Rai University of Veterinary and Animal Sciences, Hisar, 125004 Haryana India; 4 Division of Parasitology, ICAR- Indian Veterinary Research Institute, Izatnagar, Bareilly, 243122 Uttar Pradesh India

**Keywords:** Dogs, *B. vogeli*, Cytochrome b gene, Haplotype, Genetic diversity, Evolution, Genetics

## Abstract

The current study aimed at population genetic characterization of *B. vogeli* based on the cytochrome b (*cyt b*) gene sequences (≥ 685 bp) available in the GenBank. Phylogenetic trees placed all the sequences of *B. vogeli* in a single large monophyletic clade; however, it was further divided into two subclades (Bv1 and Bv2). Out of seven nucleotide variations observed between Bv1 and Bv2 subclades, four were synonymous (G92A, C170T, T488C and A659G), and three were non-synonymous (G324A, C438A and G465A) resulting in amino acid substitutions at three places (V108I, L146I and V155I). Within different *B. vogeli* populations, the nucleotide and haplotype diversities were low. The median-joining haplotype network revealed only two haplotypes (Hap_1 and Hap_2). A geographical sub-structuring was noticed in the *B. vogeli* populations, with moderate genetic differentiation (F_ST_ = 0.05000; *P* < 0.05) and a very high gene flow (Nm = 4.75) between Indian and Chinese populations. Neutrality tests and mismatch distributions for the Indian population and the overall dataset of *B. vogeli* indicated a constant population size. This study provides the first insight into the genetic characterization, population genetics and haplotype network of *B. vogeli* based on the *cyt b* gene.

## Introduction

Amid various tick-borne diseases, canine babesiosis (or piroplasmosis) is a clinically significant, emerging, and potentially life-threatening ailment of the dog population, prevailing throughout the world^1^. It is caused by intraerythrocytic apicomplexan protozoa of the genus *Babesia* (order ‒ Piroplasmida), which are transmitted by the bites of tick vectors^2,3^. On the basis of size of piroplasms during microscopic examination of blood smears, two discrete forms of *Babesia* are identified – large (2.5–5.0 μm) and small form (1.0–2.5 μm)^4^. Large canine piroplasms consist of three species, viz*., B. canis*, *B. rossi* and *B. vogeli* transmitted by *Dermacentor reticulatus* (in Europe), *Haemaphysalis elliptica* (in Africa), and *Rhipicephalus sanguineus* (in Asia, Africa, America and Australia), respectively^2^. A fourth genetically distinct ‘large’ unnamed *Babesia* sp. has been reported in a number of dogs with clinical signs from North Carolina, New Jersey and New York in the USA^5,6^. Similarly, *B. gibsoni*, *B. conradae*, *B. negevi*^7,8^ and *B. vulpes* (Syn. *Theileria annae* and *B. microti*-like piroplasm)^9^ are small forms of *Babesia* known to cause disease in dogs^10^. The geographical distribution of *Babesia* spp. depends on the existence of the competent tick vectors for dissemination.

In India, only two species, *B. vogeli* and *B. gibsoni*, have been reported to cause canine babesiosis till date^11,12^. Clinical signs of this disease are extremely variable and depend on various determinants, such as, the infecting *Babesia* species, signalment, host immunity, splenectomy and concomitant infections^2^. Severe clinical infections with *B. vogeli* typically occur in puppies. Additionally, adult dogs with concomitant infectious or non-infectious diseases can exhibit severe clinical manifestations. The disease is characterized by apathy, anemia (leading to pallor of the mucous membranes), tachypnea, tachycardia, thrombocytopenia, lymphadenomegaly, splenomegaly, inappetence to anorexia, debility, icterus, pigmenturia, and occasional death^1^. For precise diagnosis of *B. vogeli*, a holistic approach based on the history, clinical signs, blood smear examination by light microscopy, serological tests and molecular assays, is required. For better understanding of the epidemiology, pathogenesis, treatment and control of the disease, it is extremely important to confirm the species and/or sub-species/genotypes associated with canine babesiosis. The mitochondrial genes exhibit greater genetic diversity compared to the nuclear markers. The mitochondrial cytochrome b (*cyt b*) gene is a potential genetic marker which has been used for species level identification, strain differentiation, phylogenetic classification, drug resistance monitoring and the development of new diagnostic tests, leading to earlier diagnosis, treatment and better prognosis^13^. Apart from a few isolated reports based on the *18S rRNA* gene^14–18^, there is paucity of scientific literature on the genetic characterization of *B. vogeli* in dogs, and till now, there is no report on the molecular characterization and phylogenetic analysis of *B. vogeli* infecting dogs on the basis of the *cyt b* gene. Therefore, systematic studies are required to understand the cladistics and genetic diversity of *B. vogeli*. In the present study, we report the sequence, phylogenetic and haplotype analyses of *B. vogeli* based on the *cyt b* gene, along with the genetic diversity, neutrality tests and population-genetic structure of the said parasite.

## Materials and methods

### Collection of blood samples and isolation of genomic deoxyribonucleic acid (DNA)

Approximately two mL peripheral blood sample was collected from dogs (n = 21) microscopically positive for large *Babesia* spp. and exhibiting clinical signs, viz., fever, lymphadenopathy, anemia (leading to pale mucous membranes), tachypnea, tachycardia, weakness, inappetence to anorexia, tick infestation and/or history of tick infestation, over a time period of one year during May, 2022–May, 2023. The collected blood samples were immediately transferred into ethylene diamine tetra-acetic acid (EDTA) coated tubes for DNA extraction. The details of the samples included in the present study are provided in the supplementary Table 1. Ethical permission for the collection of blood samples was obtained from the Institutional Animal Ethics Committee (IAEC) of Lala Lajpat Rai University of Veterinary and Animal Sciences (LUVAS), Hisar, Haryana, India (IAEC Permission No. VCC/IAEC/2022/1679-1705 dated 17-05-2022; Agenda No. 24). All experiments were performed in accordance with the relevant guidelines and regulations, and this study adheres to the ARRIVE guidelines (https://arriveguidelines.org).

By using 200 μL of whole anticoagulated blood, the genomic DNA was extracted using the QIAamp DNA mini kit (Qiagen, Germany) following the manufacturer’s procedure. The purity^19,20^, concentration, and quality (260/280 ratio) of extracted DNA were assessed as mentioned previously^21,22^ using optical spectrophotometry (Nanodrop, Thermo Scientific). The eluted DNA was stored at − 20 °C until further use. Additionally, the isolated genomic DNA from a known negative dog and nuclease-free water served as negative and no-template controls, respectively, while the DNA from an earlier confirmed *B. vogeli*-infected puppy served as a positive control in each Polymerase Chain Reaction (PCR).

### Species level identification of the large *Babesia* spp. using single-step PCR assays

The genomic DNA isolated from blood samples (n = 21) was subjected to a PCR assay described by Duarte et al.^23^ for species level identification of large *Babesia* spp. The primers details are listed in supplementary Table 2. Amplification was performed using the reaction mixture and conditions described by the authors^23^. The amplified PCR product was visualized by electrophoresis in a 1.5% agarose gel stained with ethidium bromide under UV transillumination (Biorad, USA), as described previously^24,25^.

### Optimization of the *cyt b* gene based PCR assay and sequencing of *B. vogeli*

A self-designed *B. vogeli* primer set (Bvogeli693F 5’-TGGACTTTTCGCTATTTTCAT-3’ and Bvogeli693R 5’-AGCTCTAGATTCGACAACAAGTAT-3’) was used to amplify the partial sequence of the mitochondrial *cyt b* gene (693 bp) in this study. The PCR was carried out in a 25 µL volume containing 10–50 ng of template DNA, 12.5 µL of Phusion High-Fidelity PCR Master Mix (Thermo Scientific, USA), 1.0 µL of each forward and reverse primers (10 pmol/µL), and the remaining amount of nuclease free water. Amplification reactions were performed in a T100 Thermal Cycler (Bio-Rad, USA) with an initial denaturation at 98 °C for 1 min, followed by 35 amplification cycles (98 °C for 10 s, 64 °C for 40 s, and 72 °C for 45 s), and a final extension step at 72 °C for 10 min. The amplified PCR products were checked by 1.2% Tris–acetate-EDTA agarose gel electrophoresis, as described previously^19^. To ascertain the size of amplified PCR products, StepUp 100 bp DNA ladder (Genei, India) was used.

Thereafter, the PCR amplified product of all the isolates (n = 21) was gel purified using the MinElute Gel Extraction Kit (Qiagen, Germany), and the concentration and purity of the purified products were estimated using a Nanodrop™ 2000 spectrophotometer (Thermo Scientific, USA). Amplicons of ~ 693 bp size were submitted for custom DNA sequencing to the AgriGenome Labs Pvt. Ltd., Cochin, Kerala (India). Bidirectional Sanger sequencing was performed using the Bvogeli693F and Bvogeli693R primers. Sequence of each isolate was generated three times to rule out the sequencing error. The raw nucleotide sequences obtained after custom sequencing were analyzed as described previously^13^ using BioEdit software version 7.0.5.3^26^. Each sequence was subjected to pairwise alignment with reference sequences from the GenBank using the NCBI Basic Local Alignment Search Tool (BLASTn; https://blast.ncbi.nlm.nih.gov/Blast.cgi) for identification.

### Multiple sequence alignment and phylogenetic analyses

The NCBI BLAST program was used to acquire homologous sequences (≥ 685 bp) of the *cyt b* gene of *B. vogeli* and other related *Babesia* species available in the GenBank using the default matrix corresponding to positions eight and 693 of the gene sequence of Meerut isolate (OR577229, Uttar Pradesh, India) of *B. vogeli* generated in the current study. Smaller and truncated sequences (< 685 bp), including 10 *cyt b* sequences (MZ603871-MZ603880, Brazil) of *B. vogeli* originating from dogs (*Canis lupus familiaris*), were excluded from the analysis. A total of 30 sequences of *B. vogeli* (n = 28), *B. canis* (n = 01) and *B. ovata* (n = 01), originating from different countries, were included in the dataset (Supplementary Table 1). Out of 28 *B. vogeli* sequences, 21 were newly generated in this study.

**Fig. 1:**
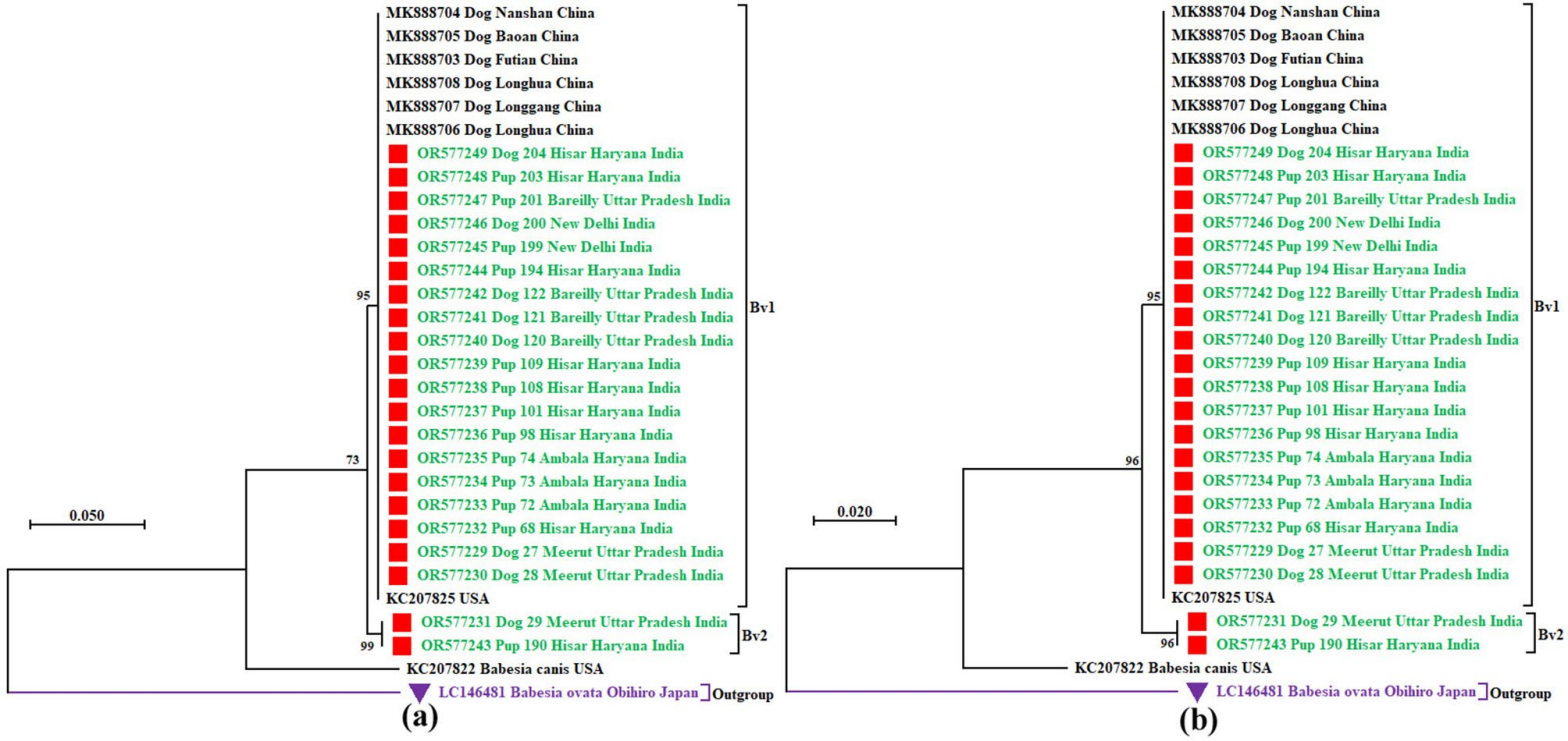
(**a**) Phylogenetic tree of *B. vogeli* based on nucleotide sequences of the *cyt b* gene constructed using the Hasegawa-Kishino-Yano model of the maximum likelihood method. A discrete Gamma distribution was used to model evolutionary rate differences among sites [3 categories (+ G, parameter = 0.5803)]. This analysis involved 30 nucleotide sequences with 686 aligned positions. (**b**) Phylogenetic tree of *B. vogeli* based on amino acid sequences of the *cyt b* gene constructed using the General Reversible Mitochondrial model of the maximum likelihood method. This analysis involved 30 amino acid sequences with 228 aligned positions. The tree is drawn to scale, with branch lengths measured in the number of substitutions per site. The taxon name of each sequence is depicted by the accession number followed by the name of the host, the place of sampling, if any, and the country of origin. The colour coding of the different sequences is as below. The red filled square as a taxon marker with a green taxon name represented the newly generated Indian *B. vogeli* sequences. The default black taxon name represented foreign *B. vogeli* and *B. canis* sequences available in the GenBank. A purple filled inverted triangle as a taxon marker with a purple taxon name represented an outgroup species.

A multiple sequence alignment was constructed using 30 sequences with the ClustalW program within MEGA-X version 10.1.7^27^, using the default settings described by Nehra et al.^28^. Evolutionary history was inferred using the Hasegawa-Kishino-Yano (HKY + G)^29^ and General Reversible Mitochondrial models^30^ of the maximum likelihood method with 1000 bootstrap replicates for nucleotide and amino acid sequences, respectively. A discrete Gamma distribution was used to model evolutionary rate differences among sites (3 categories; + G, parameter = 0.5803) for nucleotide sequences. *Babesia ovata* (LC146481, Japan) was used as an outgroup to root the tree. This analysis involved 30 nucleotide and amino acid sequences, with 686 and 228 aligned positions, respectively (Fig. 1a,b).

### Analyses of sequences, haplotype network and genetic diversity

**Fig. 2:**
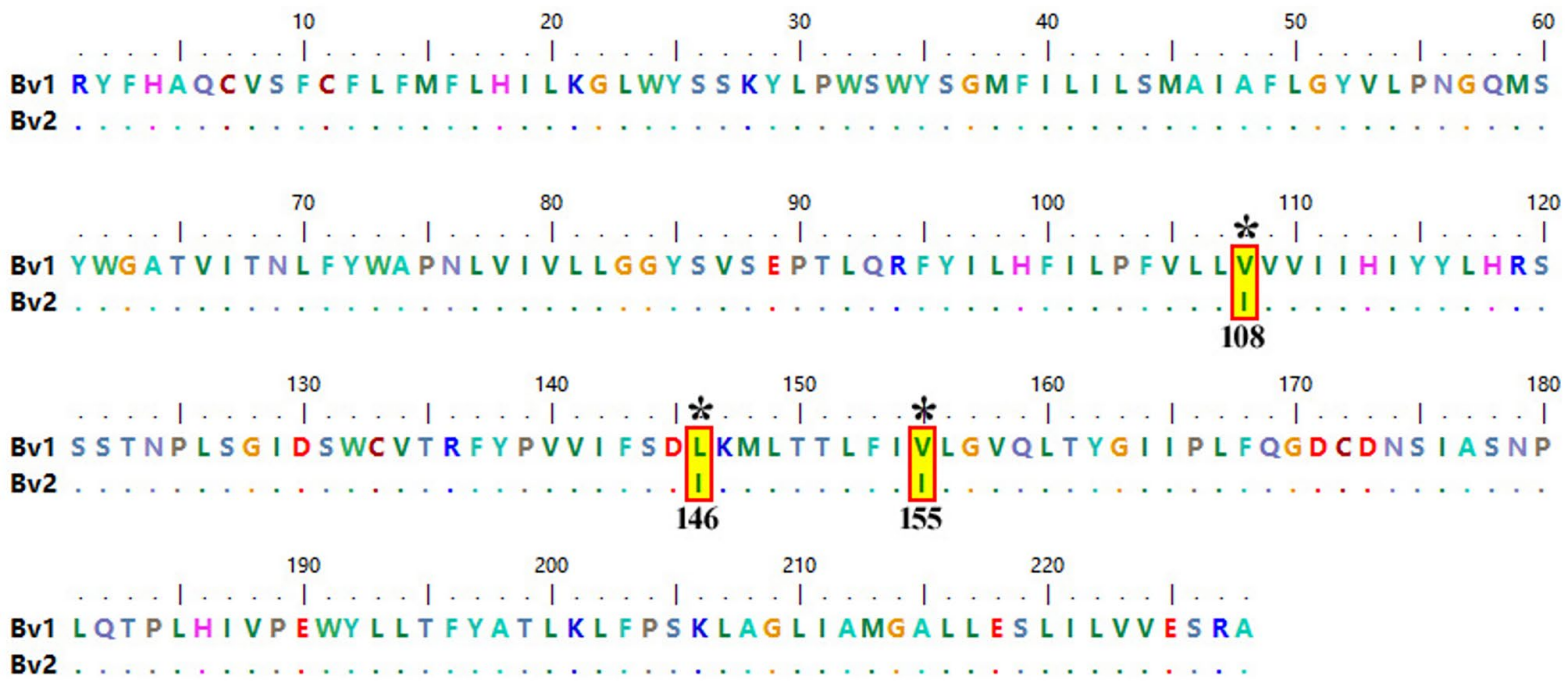
Multiple amino acid sequence alignment of the *cyt b* gene of the Bv1 and Bv2 subclades of *B. vogeli* indicated three non-synonymous mutations (V108I, L146I and V155I; marked * and shaded yellow in red boxes).

Identity, query coverage, and e-values were assessed using the BLASTn tool with default parameters and a non-redundant (nr) database available in the NCBI GenBank^31^. Comparative nucleotide (Supplementary File 1) and amino acid sequence analyses (Fig. 2) of the *cyt b* gene of *B. vogeli* isolates generated in this study were carried out with each other and with all other sequences available in the GenBank to analyze sequence variations. Percent nucleotide identity (Table 1) was computed using the MegAlign program^32^ of DNASTAR (Lasergene 6.0 package, USA). The details of *cyt b* gene sequences of Indian and foreign isolates/strains, along with their accession numbers, are tabulated in supplementary Table 1.

**Table 1:** Association attributes of *B. vogeli* based on the *cyt b* gene.

*B. vogeli* sub-clade	Percent nucleotide and amino acid identity within sub-clade	Percent nucleotide and amino acid identity between sub-clades	Closest *Babesia* species	Percent nucleotide identity with closest *Babesia* species
Bv1	100 and 100	99.0 and 98.7	*B. canis*	89.9
Bv2	100 and 100	99.0 and 98.7	*B. canis*	89.9

**Fig. 3:**
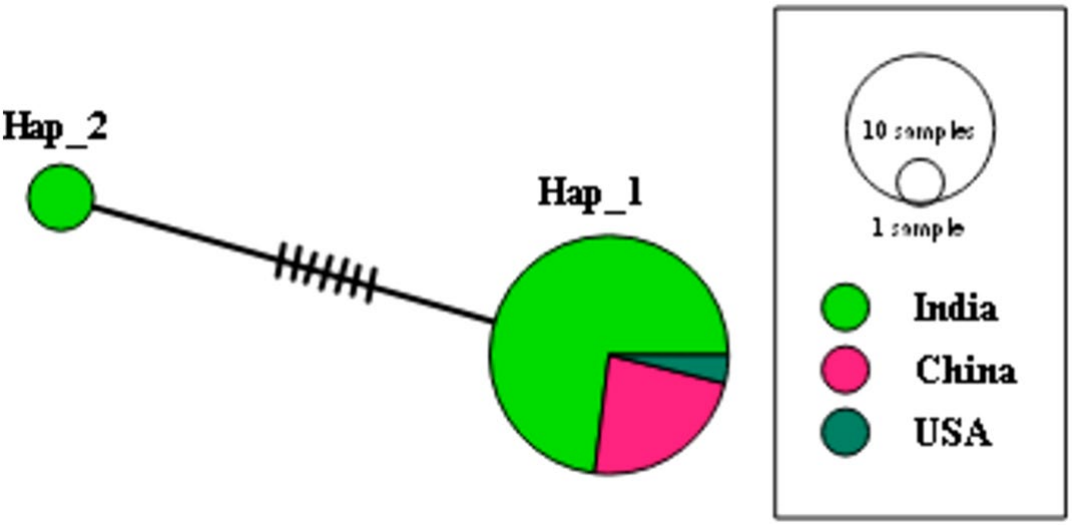
Median-joining haplotype network of the *cyt b* gene of *B. vogeli* constructed using PopArt. Each circle represents a unique haplotype and the size of the circle is proportional to the number of sequences included. Nucleotide variations are denoted by the hatch marks across the lines connecting the haplotypes with each bar representing a single nucleotide variation. A colour code to the country of origin is given.

The relationship between *B. vogeli* haplotypes based on the country of origin was estimated using median joining haplotype network analysis in PopART^33^, as described by Nehra et al.^13,34^. In total, 28 *cyt b* sequences of *B. vogeli* from three countries were included (Fig. 3; Table 2).

**Table 2:** Various haplotypes of *B. vogeli* identified in the current study based on the *cyt b* gene.

Haplotype	No. of sequences	Accession number (Place of origin)	Country
Hap_1	26	OR577229 (Meerut, Uttar Pradesh, India ); OR577230 (Meerut, Uttar Pradesh, India); OR577232 (Hisar, Haryana, India); OR577233 (Ambala, Haryana, India); OR577234 (Ambala, Haryana, India); OR577235 (Ambala, Haryana, India); OR577236 (Hisar, Haryana, India); OR577237 (Hisar, Haryana, India); OR577238 (Hisar, Haryana, India); OR577239 (Hisar, Haryana, India); OR577240 (Bareilly, Uttar Pradesh, India); OR577241 (Bareilly, Uttar Pradesh, India); OR577242 (Bareilly, Uttar Pradesh, India); OR577244 (Hisar, Haryana, India); OR577245 (New Delhi, India); OR577246 (New Delhi, India); OR577247 (Bareilly, Uttar Pradesh, India); OR577248 (Hisar, Haryana, India); OR577249 (Hisar, Haryana, India); MK888706 (Longhua, China); MK888707 (Longgang, China); MK888708 (Longhua, China); MK888703 (Futian, China); MK888704 (Nanshan, China); MK888705 (Baoan, China); KC207825 (USA)	India, China and USA
Hap_2	02	OR577231 (Meerut, Uttar Pradesh, India); OR577243 (Hisar, Haryana, India)	India

The genetic diversity indices (Table 3) based on the *cyt b* gene of *B. vogeli*, viz*.,* number of variable sites (S), total number of mutations (η), average number of nucleotide differences (k), number of haplotypes (h), nucleotide diversity (π) and haplotype diversity (Hd), were estimated for each country and the overall dataset using DnaSP ver. 6.12.03^35^.

**Table 3:** Haplotype/nucleotide diversity and neutrality tests of *B. vogeli* populations based on the *cyt b* gene.

Name of statistical data	India	China	Total
Number of sequences (n)	21	6	28
Number of variable sites (S)	7	0	7
Total number of mutations (η)	7	0	7
Nucleotide diversity (π) ± SD	0.00185 ± 0.00107	0.00000 ± 0.00000	0.00140 ± 0.00085
Average number of nucleotide differences (k)	1.267	0.000	0.963
Number of haplotypes (h)	2	1	2
Haplotype diversity (Hd) ± SD	0.181 ± 0.104	0.000 ± 0.000	0.138 ± 0.084
Tajima's D	− 1.13144^NS^	–	− 1.39614^NS^
Fu and Li's F	0.69783^NS^	–	0.55894^NS^
Ramos-Onsins and Rozas’ R2	0.0905	–	0.0688

A single representative sequence from USA was not included as this analysis required a minimum of two sequences from one country.

^N^^S^ Indicates non-significant (*P* > 0.10).

### Population-genetic structure and demographic history

Genetic differences were estimated using statistics based on haplotypes (Hs), nucleotide sequences (Ks), and other parameters which reflect gene flow (Nm), viz*.,* average number of nucleotide differences in pairs (Kxy), genetic differentiation index based on the frequency of haplotypes (Gst), nucleotide-based statistics (Nst), nucleotide substitutions per site (Dxy) and net nucleotide substitutions per site (Da), using DnaSP ver. 6.12.03^34,35^. Gene flow (Nm) and the pairwise genetic distance (F_ST_) between populations were computed to determine the genetic differentiation and population-genetic structure of *B. vogeli* using Arlequin 3.5.2^36^. For AMOVA, the F_ST_ was computed using the Tajima-Nei substitution model with 1000 permutations to test its significance.

The neutrality tests (Tajima's D, Fu and Li's F, and Ramos-Onsins and Rozas’ R2), Harpending’s raggedness index (rg)^37^, mean absolute error (MAE), and the mismatch-distribution analysis were performed to ascertain the demographic history using DnaSP ver. 6.12.03^35,38^.

### Secondary structure and homology modeling

Various protein prediction programs, viz*.,* PSIPRED^39^, NetNGlyc^40^, NetOGlyc^41^, SignalP 5.1^42^, and tools form ExPASy server (https://www.expasy.org/resources/swiss-model), were used to predict the secondary structure and homology modeling of the consensus amino acid sequence of the *cyt b* gene of the newly generated isolates of *B. vogeli*. An automated homology model of the *cyt b* was predicted using SWISS‐MODEL^43,44^ based on the structure of the *cyt b* protein of *B. gibsoni* (UniProt ID—A0A649UJK2).

## Results

### Species level identification of the large *Babesia* spp. using single-step PCR assays

All the samples (n = 21) included in the present study exhibited a PCR amplicon of ~ 600 bp size, as per the PCR assay described by Duarte et al.^23^, which indicated the presence of *B. vogeli* infection in all dogs (Supplementary Fig. 1). None of the dogs were found to be infected with *B. canis* and *B. rossi*.

### PCR amplification and sequencing of the *cyt b* gene of *B. vogeli*

The standardized PCR assay based on the *cyt b* gene of *B. vogeli* produced amplicons of ~ 693 bp (Supplementary Fig. 2) with all the newly generated isolates (n = 21), which was further confirmed by sequencing. The analyzed sequences of all the isolates were submitted to the GenBank and the details of the accession numbers (OR577229–OR577249) obtained are listed in the supplementary Table 1.

### Multiple sequence alignment and phylogenetic analysis of *B. vogeli* based on the *cyt b* gene

The best substitution models for construction of the maximum likelihood trees were found to be the Hasegawa-Kishino-Yano and General Reversible Mitochondrial models for nucleotide and amino acid datasets, respectively. Phylogenetic trees constructed using nucleotide and amino acid sequences of the *cyt b* gene of *B. vogeli* and other related *Babesia* species available in the GenBank are presented in Fig. 1a and b, respectively. Both phylograms placed all the sequences of *B. vogeli* in one large monophyletic clade; however, it was further divided into two small subclades with high bootstrap values (> 95%), hitherto designated as Bv1 and Bv2. Out of the total 28 sequences, subclades Bv1 and Bv2 encompassed 26 and two sequences, respectively. All the isolates from each country clustered together in close vicinity, indicating a geographical sub-structuring in the *B. vogeli* populations.

### Analyses of sequences, haplotype network and genetic diversity

All the nucleotide and amino acid sequences of the *cyt b* gene of *B. vogeli* (n = 28) included in the present study exhibited 99.0–100% and 98.7–100% similarity among themselves, respectively. The Bv1 and Bv2 subclades revealed 99.0 and 100% nucleotide, and 98.7 and 100% amino acid similarity between and within them, respectively (Table 1). Furthermore, the newly generated Indian sequences (n = 21) originating from Delhi, Haryana, and Uttar Pradesh displayed 99.0–100% and 98.7–100% nucleotide and amino acid identity, respectively, between them. The nucleotide sequence alignment of the *cyt b* gene of *B. vogeli* revealed single nucleotide substitutions at seven places (G92A, C170T, G324A, C438A, G465A, T488C and A659G). No nucleotide variations were observed within the Bv1 and Bv2 subclades, but sequence variations were observed at seven places (G92A, C170T, G324A, C438A, G465A, T488C and A659G) between them (Supplementary File 1). Out of these mutations, four were synonymous (G92A, C170T, T488C and A659G), and three were non-synonymous (G324A, C438A and G465A), resulting in amino acid substitutions at three places (V108I, L146I and V155I; Fig. 2).

In total, 28 sequences of the mitochondrial *cyt b* gene were used to assess the relationship between *B. vogeli* haplotypes and their country of origin. The median-joining haplotype network of *B. vogeli* based on the *cyt b* gene revealed only two haplotypes (Hap_1 and Hap_2), both of which exhibited a difference of seven nucleotides from each other (Fig. 3). Hap_1 and Hap_2 consisted of twenty-six and two sequences, respectively (Table 2). India, and China and the United States of America (USA) exhibited two and one haplotypes, respectively (Table 3). The major haplotype, Hap_1, was recorded from India, China and the USA, while the minor haplotype (Hap_2), represented by the two newly generated sequences (OR577231 and OR577243), was documented from India only. Therefore, both haplotypes were present in India.

The summary of genetic diversity indices of different *B. vogeli* populations based on the *cyt b* gene is presented in Table 3. However, these parameters could not be determined for the *B. vogeli* population originating from the USA, as only one sequence of USA origin was included in the analyses. Along the 686 bp alignment, seven nucleotide mutations were detected at seven places. Within different *B. vogeli* populations, the nucleotide diversity (π; ranging from 0.00000 ± 0.00000 to 0.00185 ± 0.00107) and the haplotype diversity (Hd; ranging from 0.000 ± 0.000 to 0.181 ± 0.104) were low. The haplotype diversity of the Indian population and the combined dataset was less than 0.2, suggesting a low level of haplotype diversity. The average number of nucleotide differences between any two sequences in the Indian population (k = 1.267) and the overall dataset (k = 0.963) was very low, indicating extensive sequence conservation within the *cyt b* gene. Notably, the Chinese population exhibited complete sequence homogeneity, with no variations in the *cyt b* gene.

### Population-genetic structure and demographic history

Gene flow and genetic differentiation indices between the Indian and Chinese *B. vogeli* populations based on the *cyt b* gene are tabulated in Table 4. The pairwise genetic distance between these two populations (F_ST_ = 0.05000), which was statistically significant (P < 0.05), indicated moderate genetic differentiation (0.05 to 0.15). Contrary to the F_ST_ index, the value of gene flow (Nm) between these two *B. vogeli* populations was very high (Nm = 4.75; Table 4). All sequence variations were found within *B. vogeli* populations upon AMOVA analysis and extensive sequence homogeneity was registered between populations (Supplementary Table 3).

**Table 4:** Gene flow and genetic differentiation indices between *B. vogeli* populations based on the *cyt b* gene.

Population 1	Population 2	Hs	Ks	Kxy	Gst	Nst	F_ST_	Nm	Dxy	Da
India	China	0.14948	0.98519	0.66667	0.02746	0.05000	0.05000*	4.75	0.00097	0.00005

Hs: Hudson’s haplotype-based statistics; Ks: Hudson’s nucleotide sequence-based statistics^45^; Kxy: Average proportion of nucleotide differences between *B. vogeli* populations; Gst: Genetic differentiation index based on the frequency of haplotypes; Nst: Nucleotide-based statistics^46^; F_ST_: Pairwise genetic distance^45^; Dxy: Average number of nucleotide substitutions per site between *B. vogeli* populations; Da: Number of net nucleotide substitutions per site between *B. vogeli* populations; *Indicates statistical significance (*P* < 0.05).

**Fig. 4:**
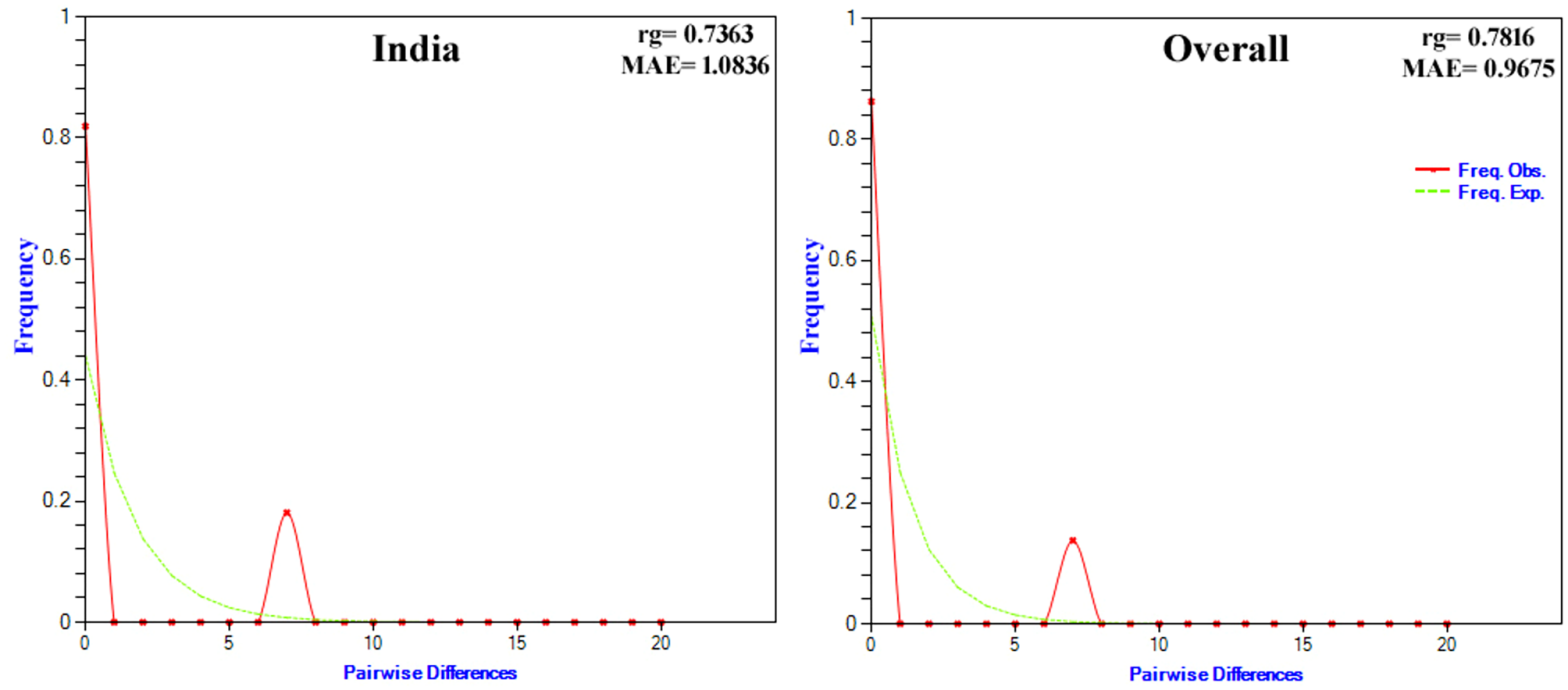
Representation of the observed and expected mismatch-distribution, Harpending’s raggedness index (rg) and mean absolute error (MAE) of the Indian *B. vogeli* population and the overall dataset on the basis of the *cyt b* gene.

The summary of neutrality tests, viz*.,* Tajima’s D, Fu and Li’s F, and Ramos-Onsins and Rozas’ R2, of the *B. vogeli* populations originating from India and China and the overall dataset based on the *cyt b* gene is depicted in Table 3. The non-significant negative values of Tajima’s D, non-significant positive values of Fu and Li’s F, small positive values of Ramos-Onsins and Rozas’ R2 (Table 3), and the bimodal mismatch distributions (Fig. 4) for the Indian population and overall dataset of *B. vogeli* implied a constant population size.

### Secondary structure and homology modeling

**Fig. 5:**
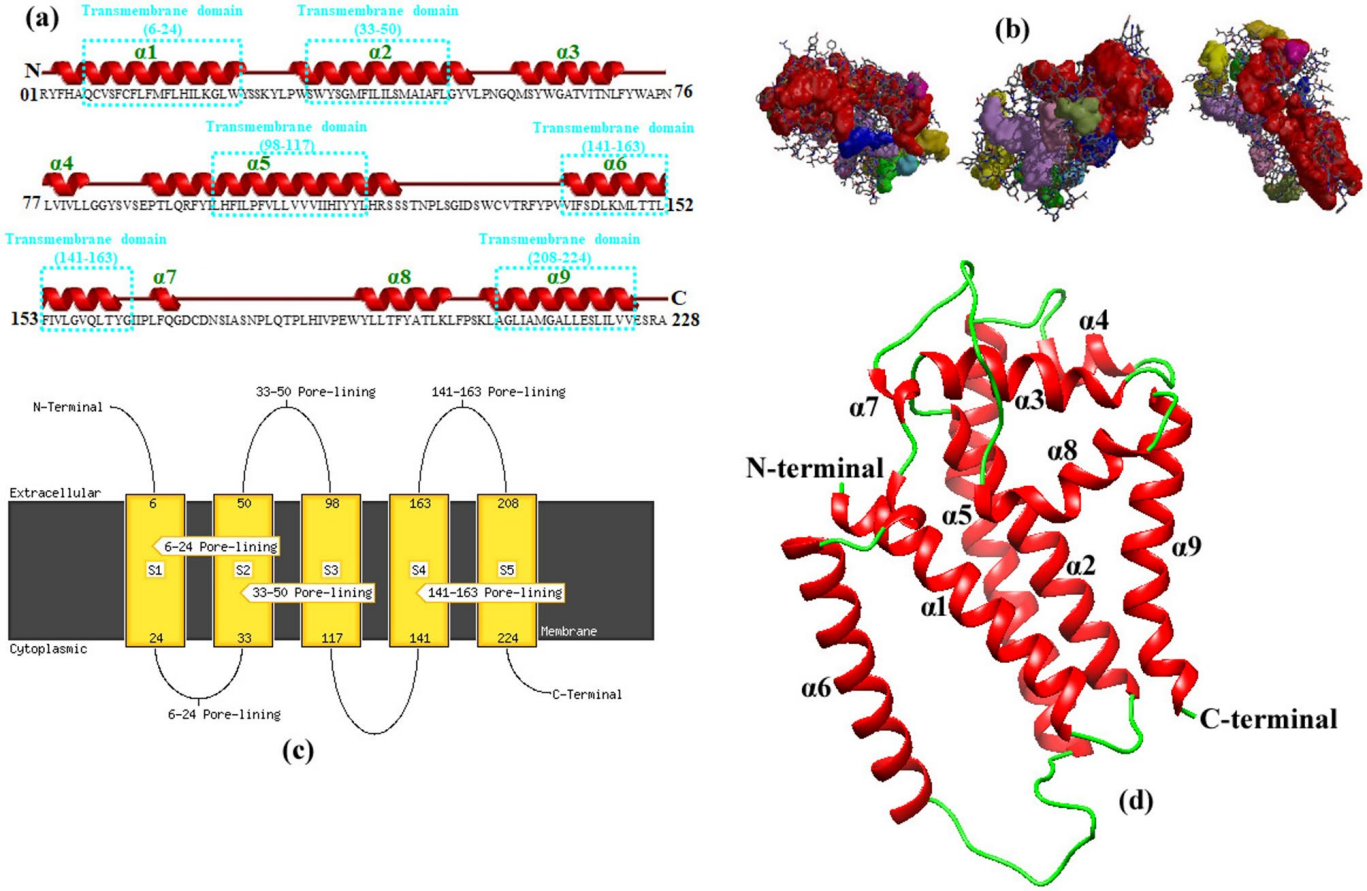
Protein characteristics and homology modeling of the partial *cyt b* protein of *B. vogeli*. (**a**) Secondary structure of 228 amino acids *cyt b* protein contained nine alpha helices (red; numbered in green) as predicted by PSIPRED. (**b**) Surface clefts are depicted as solid-coloured regions according to volume, with the largest shown in red, and it represents the protein's binding site. (**c**) It is composed of three extracellular domains (_1_R-A_5_, _51_G-I_97_, and _164_I-L_207_,), five transmembrane domains (_6_Q-W_24_, _33_S-L_50_, _98_L-L_117_, _141_ V-G_163_ and _208_A-V_224_) and three cytoplasmic domains (_25_Y-W_32_, _118_H-V_140_ and _225_E-A_228_). (**d**) Ribbon representation of homology model of the *cyt b* protein produced using automated homology prediction by SWISS-MODEL server by utilizing the cytochrome b protein of *B. gibsoni* (UniProt ID—A0A649UJK2) as template. Alpha helices and loops/coils are represented in red and green colors, respectively. The abbreviations used are as follows: α, alpha helix; N – N-terminus; C – C-terminus.

The partial cytochrome b protein characterized in this study is a 26.04 kDa protein containing 228 amino acids with 8.158 isoelectric point (pI). Analysis of secondary structure predicted the presence of nine alpha helices and no beta sheet. Schematic diagram of the secondary structure and homology model of the consensus sequence of cytochrome b protein of the Indian isolates is illustrated in Fig. 5. The protein features three extracellular domains (_1_R-A_5_, _51_G-I_97_, and _164_I-L_207_,), five transmembrane domains (_6_Q-W_24_, _33_S-L_50_, _98_L-L_117_, _141_ V-G_163_ and _208_A-V_224_) and three cytoplasmic domains (_25_Y-W_32_, _118_H-V_140_ and _225_E-A_228_; Fig. 5a,c). Notably, it neither contained a disulphide bond nor glycosylation sites and a signal peptide. The clefts envisaged to be present on the surface of the cytochrome b protein are reproduced as solid-coloured regions in Fig. 5b with their size varying according to volume. The largest cleft, displayed in red, represents the binding site. The 3‐D structure demonstrating the ribbon representation of the homology model of the 228 amino acid residues of the partial cytochrome b protein of *B. vogeli*, using the cytochrome b protein of *B. gibsoni* (UniProt ID—A0A649UJK2) as template, is depicted in Fig. 5d.

## Discussion

Favourable environmental conditions existing in different agro-climatic zones of tropical India facilitate the perpetuation of the ectoparasitic life cycle and transmission of various vector-borne diseases^47^. As *B. vogeli* is transmitted by *Rhipicephalus sanguineus*, which is ubiquitous in distribution, transmission of infection can occur easily to susceptible hosts^11^. There is no report on population genetic characterization of *B. vogeli* infecting dogs in India. In the current study, we conducted the sequence and phylogenetic analyses, population genetic diversity, genetic structure, and haplotype network analyses of *B. vogeli*, based on the *cyt b* gene sequences in the GenBank for the first time.

Mitochondrial markers are becoming the preferred choice for studying genetic diversity and phylogenetic relationships due to their higher sequence variability compared to the nuclear markers^48^. Recently, this genetic marker has been used for characterization^49^, taxonomic classification^50^, identification of new species^51^, and phylogenetic analysis of apicomplexan parasites^49^. That is why, the mitochondrial *cyt b* gene of *B. vogeli* was targeted in the present study.

The maximum likelihood trees constructed using the nucleotide and amino acid sequences of the *cyt b* gene grouped all the true *B. vogeli* sequences into a single major clade, and the sequences originating from one country were almost located close to each other. This signified the presence of geographical sub-structuring in the *B. vogeli* population. Similar results have recently been reported with the *cyt b* gene of *T. annulata*^13^.

Sequence analysis displayed an extensive sequence conservation among the *cyt b* sequences of *B. vogeli*. It reinforced the previous findings that the *cyt b* gene sequences of apicomplexan parasites are highly conserved^13,21,51^. Due to the degeneracy of codons, the Bv1 and Bv2 subclades of *B. vogeli* based on the *cyt b* gene differing by seven nucleotides exhibited a variation of only three amino acids.

Haplotype network analysis is often a more effective and informative method for studying genetic variations within a species than phylogenetic trees, which are better suited for revealing evolutionary relationships between distantly related organisms. Haplotype networks focus on subtle genetic variations, such as single nucleotide polymorphisms, between closely related individuals. This makes haplotype networks a powerful tool for studying genetic diversity even in closely related groups^52^. Analysis of the *cyt b* gene of *B. vogeli* revealed a geographically widespread ancestral haplotype. Haplotype network analysis identified only two haplotypes. This was consistent with the findings of sequence and phylogenetic analyses. Interestingly, only one haplotype (Hap_1) was found in several countries, while the other (Hap_2) was unique to India. This finding suggested that Hap_1 may be the ancestral haplotype due to its wider presence in the dog population^13,53^. The haplotype diversity of *B. vogeli* indicates genetic changes, and the transmission of *Babesia* by ticks suggests that some variations may be more easily transmitted than others, potentially influencing the regional haplotype distribution^54^.

A very low haplotype and nucleotide diversities of the overall and Indian datasets of *B. vogeli* indicated a very low level of genetic diversity among different populations of *B. vogeli*, which was due to sequence conservation observed between populations. Similar results have recently been reported for *B. gibsoni* on the basis of the *18S rRNA* gene^53^. It signified the presence of only negligible differences (seven nucleotides) between haplotypes, which was also evident in the haplotype network. The limited haplotype diversity of *B. vogeli* may be due to the small population size in this study, as the *cyt b* sequences currently available in the GenBank database are very limited, and the population size is a key factor affecting genetic diversity. However, the authors believe that it can possibly increase over time with the addition of more *cyt b* sequences of *B. vogeli* from different geographical regions. The underlying reasons for spontaneous generation of mutations in the cytochrome b gene could be due to the less efficient proofreading by the mitochondrial DNA polymerase^55^, multi-copy number^56^ and the increased mitochondrial DNA mutagenesis due to the generation of hydroxyl radicals in the mitochondrial respiration chain^57^.

The *B. vogeli* populations between India and China exhibited moderate genetic differentiation (F_ST_ = 0.05000; *P* < 0.05) with a very high gene flow (Nm = 4.75) between them, meaning the populations exhibit some genetic differences but still share a significant amount of genetic material due to frequent exchange of genes. These results were consistent with a previous study of *T. annulata*^13^. High gene flow between India and China may be attributed to a surge in the international movement of humans with asymptomatic pet dogs as companions and/ or for commercial purposes^58^. In addition, the environmental changes promoting extension of the tick vectors have tremendously contributed to their pervasive dissemination and fast expansion, with the extension of *Rhipicephalus sanguineus* to new regions. Migratory birds can also carry ticks and tick-borne diseases between breeding and wintering areas^59^. In addition, the horizontal gene transfer in *B. vogeli* can be ascribed due to iatrogenic transmission, and migration of infected dogs and ticks^2^. Excessive gene flow between populations can decline, delay or arrest the process of genetic differentiation^34,60^. Collectively, there are many factors, viz*.,* transmission intensity, parasite-host coevolution, geographic and ecological segregation, and selective pressure, which can affect the genetic structure of an organism^53^.

The existing neutral theory has limitations in explaining the mechanisms and patterns of genetic diversity, as it underestimates the impact of selection, ignores non-neutral mutations, fails to explain high genetic diversity, neglects genomic context (including linkage and epistasis), and overlooks epigenetic factors^61^. The level of polymorphism within a population is influenced by the effective population size and neutral mutation rate^62^. However, polymorphism levels vary across genes and regions due to: (a) different functional constraints, leading to varying neutral mutation rates; (b) background selection, which reduces effective population size and alters neutral polymorphism levels; and (c) regional differences in recombination intensity and deleterious mutation production, causing drastic changes in neutral polymorphism^63^. This highlights the need for a more comprehensive understanding of genetic diversity, considering factors beyond the neutral theory.

The neutrality indices and mismatch distribution for the Indian population and overall dataset suggest a constant population size. To detect the population growth for small and large sample sizes, Ramos-Onsins and Rozas’ R_2_, and Fu’s F_s_, respectively, are the best statistical tests^64^. Since these tests analyze different aspects of mutations in the populations under investigation, it is important to use multiple neutrality tests together, as each one has its own advantages and disadvantages.

Genetic characterization of the *cyt b* gene established it as a valuable genetic marker for studying the genetic diversity, evolution, and relationship among isolates. As the dataset created included sequences of sufficient length from only three countries (India, China, and the USA) due to lack of their availability from other countries in the nucleotide databases, it represents a limitation of the present study. Further studies are necessary for a better understanding of the genetic diversity of *B. vogeli* prevalent in other regions of India as well as from other countries of different continents. Additionally, the impact of human activities and/or migration of birds on the spread of *B. vogeli* needs to be explored.

## Conclusions

This study provides the first insight into the genetic characterization, population genetics and haplotype network of *B. vogeli* based on the *cyt b* gene. Notably, the phylogenetic analysis identified two distinct sub-clades within *B. vogeli*. Consistent with this finding, the haplotype network revealed only two predominant haplotypes. A very low level of genetic diversity was recorded among *B. vogeli* populations from different geographic locations. Furthermore, demographic analyses suggest that *B. vogeli* has maintained a stable population size over time, with no significant fluctuations.

## Supplementary Information

Supplementary Figures.

Supplementary Information 1.

Supplementary Table 1.

Supplementary Table 2.

Supplementary Table 3.

## Data Availability

Nucleotide and amino acid sequence data reported in this paper are available in the GenBank database. Additional data is provided within the supplementary files.
