# Supplementary material for: Population genetics of Babesia vogeli based on the mitochondrial cytochrome b gene
Source: Sci Rep. 2024 Sep 20;14:21975. doi: 10.1038/s41598-024-72572-z (PMC11415385; doi:10.1038/s41598-024-72572-z)
Supplement: Supplementary file 1 — Supplementary Figures. [file 41598_2024_72572_MOESM1_ESM.docx]

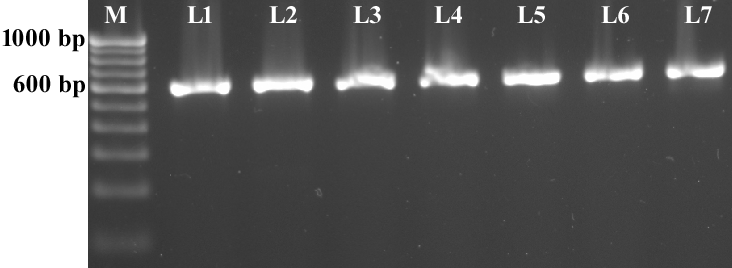


**Supplementary Figure 1a.** PCR amplification of the *18S rRNA* gene of *B. vogeli* indicated the presence of ~600 bp amplicon in all the dogs [M: StepUp 100 bp DNA ladder (Genei, India); Lanes 1-8: Positive test samples]; cropped image.


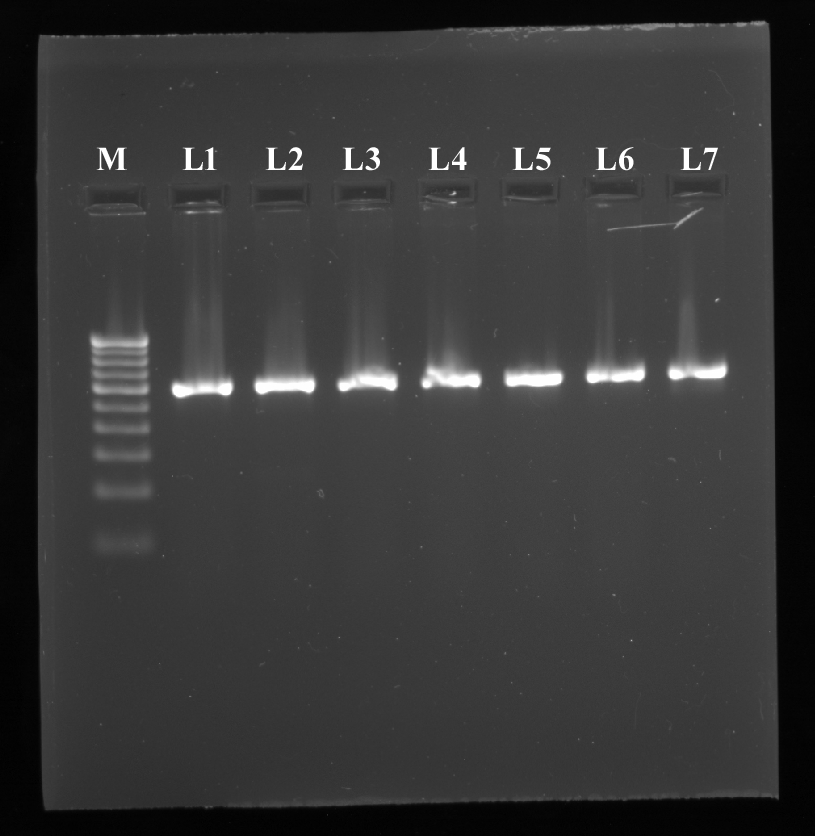


**Supplementary Figure 1b.** PCR amplification of the *18S rRNA* gene of *B. vogeli* indicated the presence of ~600 bp amplicon in all the dogs [M: StepUp 100 bp DNA ladder (Genei, India); Lanes 1-8: Positive test samples]; original image.


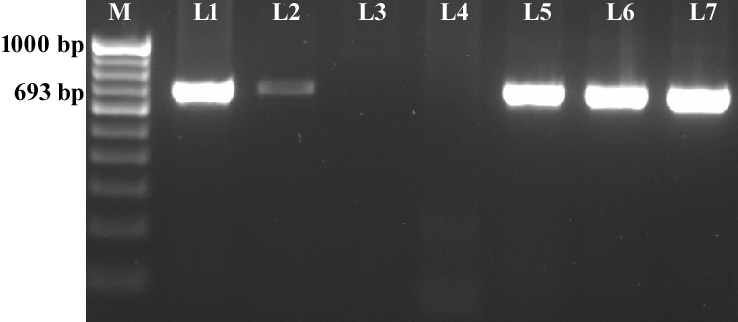


**Supplementary Figure 2a.** PCR amplification of the *cyt b* gene of *B. vogeli* indicated the presence of ~693 bp amplicon in all the dogs [M: StepUp 100 bp DNA ladder (Genei, India); Lane 1: Positive template control; Lanes 2, 5-7: Positive test samples; Lane 3: No template control; Lane 4: Negative template control]; cropped image.


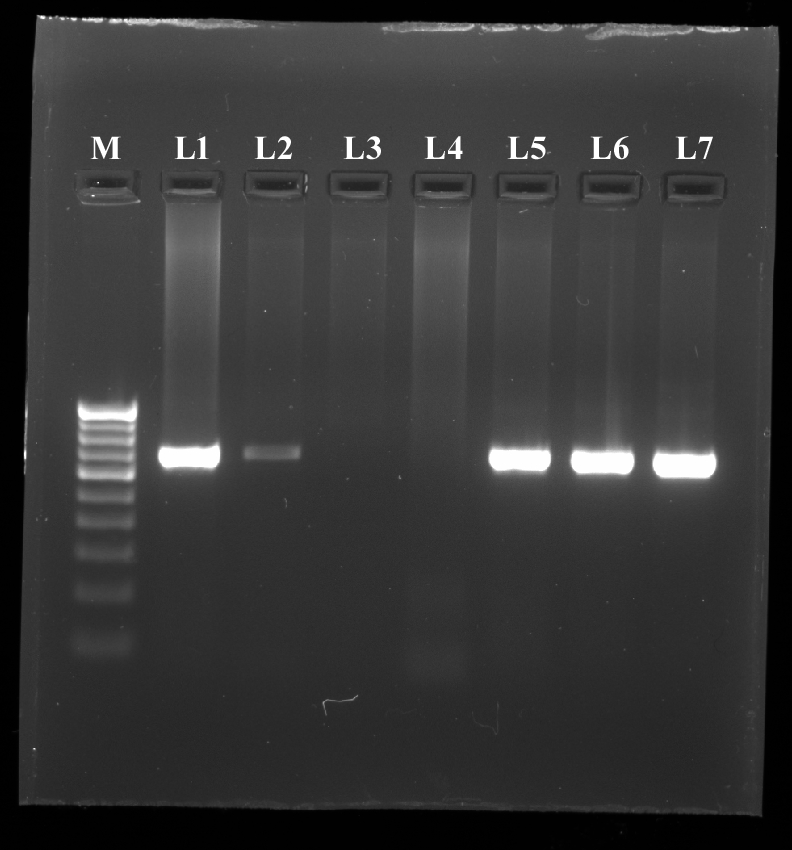


**Supplementary Figure 2b.** PCR amplification of the *cyt b* gene of *B. vogeli* indicated the presence of ~693 bp amplicon in all the dogs [M: StepUp 100 bp DNA ladder (Genei, India); Lane 1: Positive template control; Lanes 2, 5-7: Positive test samples; Lane 3: No template control; Lane 4: Negative template control]; original image.
