# Supplementary material for: Population genetics of Babesia vogeli based on the mitochondrial cytochrome b gene
Source: Sci Rep. 2024 Sep 20;14:21975. doi: 10.1038/s41598-024-72572-z (PMC11415385; doi:10.1038/s41598-024-72572-z)
Supplement: Supplementary file 2 — Supplementary Information 1. [file 41598_2024_72572_MOESM2_ESM.pdf]

```
    TTCGCTATTTTCATGCACAATGTGTGTCTTTTTGCTTTTTATTTATGTTTTTACATATATTAAAAGGATTATGGTATTCT Majority
             10        20        30        40        50        60        70        80
1   ................................................................................ KC207825 USA.seq
1   ................................................................................ MK888703 Dog Futian China.seq
1   ................................................................................ MK888704 Dog Nanshan China.seq
1   ................................................................................ MK888705 Dog Baoan China.seq
1   ................................................................................ MK888706 Dog Longhua China.seq
1   ................................................................................ MK888707 Dog Longgang China.seq
1   ................................................................................ MK888708 Dog Longhua China.seq
1   ................................................................................ OR577229 Dog 27 Meerut Uttar Pradesh In
1   ................................................................................ OR577230 Dog 28 Meerut Uttar Pradesh In
1   ................................................................................ OR577232 Pup 68 Hisar Haryana India.seq
1   ................................................................................ OR577233 Pup 72 Ambala Haryana India.se
1   ................................................................................ OR577234 Pup 73 Ambala Haryana India.se
1   ................................................................................ OR577235 Pup 74 Ambala Haryana India.se
1   ................................................................................ OR577236 Pup 98 Hisar Haryana India.seq
1   ................................................................................ OR577237 Pup 101 Hisar Haryana India.se
1   ................................................................................ OR577238 Pup 108 Hisar Haryana India.se
1   ................................................................................ OR577239 Pup 109 Hisar Haryana India.se
1   ................................................................................ OR577240 Dog 120 Bareilly Uttar Pradesh
1   ................................................................................ OR577241 Dog 121 Bareilly Uttar Pradesh
1   ................................................................................ OR577242 Dog 122 Bareilly Uttar Pradesh
1   ................................................................................ OR577244 Pup 194 Hisar, Haryana India.s
1   ................................................................................ OR577245 Pup 199 New Delhi India.seq
1   ................................................................................ OR577246 Dog 200 New Delhi India.seq
1   ................................................................................ OR577247 Pup 201 Bareilly Uttar Pradesh
1   ................................................................................ OR577248 Pup 203 Hisar Haryana India.se
1   ................................................................................ OR577249 Dog 204 Hisar Haryana India.se
1   ................................................................................ OR577231 Dog 29 Meerut Uttar Pradesh In
1   ................................................................................ OR577243 Pup 190 Hisar Haryana India.se

    AGTAAATATTTGCCATGGTCTTGGTATTCAGGAATGTTTATTCTTATTTTAAGTATGGCTATAGCTTTCTTAGGTTATGT Majority
             90       100       110       120       130       140       150       160
81  ................................................................................ KC207825 USA.seq
81  ................................................................................ MK888703 Dog Futian China.seq
81  ................................................................................ MK888704 Dog Nanshan China.seq
81  ................................................................................ MK888705 Dog Baoan China.seq
81  ................................................................................ MK888706 Dog Longhua China.seq
81  ................................................................................ MK888707 Dog Longgang China.seq
81  ................................................................................ MK888708 Dog Longhua China.seq
81  ................................................................................ OR577229 Dog 27 Meerut Uttar Pradesh In
81  ................................................................................ OR577230 Dog 28 Meerut Uttar Pradesh In
81  ................................................................................ OR577232 Pup 68 Hisar Haryana India.seq
81  ................................................................................ OR577233 Pup 72 Ambala Haryana India.se
81  ................................................................................ OR577234 Pup 73 Ambala Haryana India.se
81  ................................................................................ OR577235 Pup 74 Ambala Haryana India.se
81  ................................................................................ OR577236 Pup 98 Hisar Haryana India.seq
81  ................................................................................ OR577237 Pup 101 Hisar Haryana India.se
81  ................................................................................ OR577238 Pup 108 Hisar Haryana India.se
81  ................................................................................ OR577239 Pup 109 Hisar Haryana India.se
81  ................................................................................ OR577240 Dog 120 Bareilly Uttar Pradesh
81  ................................................................................ OR577241 Dog 121 Bareilly Uttar Pradesh
81  ................................................................................ OR577242 Dog 122 Bareilly Uttar Pradesh
81  ................................................................................ OR577244 Pup 194 Hisar, Haryana India.s
81  ................................................................................ OR577245 Pup 199 New Delhi India.seq
81  ................................................................................ OR577246 Dog 200 New Delhi India.seq
81  ................................................................................ OR577247 Pup 201 Bareilly Uttar Pradesh
81  ................................................................................ OR577248 Pup 203 Hisar Haryana India.se
81  ................................................................................ OR577249 Dog 204 Hisar Haryana India.se
81  ...........A.................................................................... OR577231 Dog 29 Meerut Uttar Pradesh In
81  ...........A.................................................................... OR577243 Pup 190 Hisar Haryana India.se

    TCTTCCAAACGGACAGATGAGTTACTGGGGAGCAACTGTTATAACAAATTTGTTTTATTGGGCTCCAAACTTAGTTATAG Majority
            170       180       190       200       210       220       230       240
161 ................................................................................ KC207825 USA.seq
161 ................................................................................ MK888703 Dog Futian China.seq
161 ................................................................................ MK888704 Dog Nanshan China.seq
161 ................................................................................ MK888705 Dog Baoan China.seq
161 ................................................................................ MK888706 Dog Longhua China.seq
161 ................................................................................ MK888707 Dog Longgang China.seq
161 ................................................................................ MK888708 Dog Longhua China.seq
161 ................................................................................ OR577229 Dog 27 Meerut Uttar Pradesh In
161 ................................................................................ OR577230 Dog 28 Meerut Uttar Pradesh In
161 ................................................................................ OR577232 Pup 68 Hisar Haryana India.seq
161 ................................................................................ OR577233 Pup 72 Ambala Haryana India.se
161 ................................................................................ OR577234 Pup 73 Ambala Haryana India.se
161 ................................................................................ OR577235 Pup 74 Ambala Haryana India.se
161 ................................................................................ OR577236 Pup 98 Hisar Haryana India.seq
161 ................................................................................ OR577237 Pup 101 Hisar Haryana India.se
161 ................................................................................ OR577238 Pup 108 Hisar Haryana India.se
161 ................................................................................ OR577239 Pup 109 Hisar Haryana India.se
161 ................................................................................ OR577240 Dog 120 Bareilly Uttar Pradesh
161 ................................................................................ OR577241 Dog 121 Bareilly Uttar Pradesh
161 ................................................................................ OR577242 Dog 122 Bareilly Uttar Pradesh
161 ................................................................................ OR577244 Pup 194 Hisar, Haryana India.s
161 ................................................................................ OR577245 Pup 199 New Delhi India.seq
161 ................................................................................ OR577246 Dog 200 New Delhi India.seq
161 ................................................................................ OR577247 Pup 201 Bareilly Uttar Pradesh
161 ................................................................................ OR577248 Pup 203 Hisar Haryana India.se
161 ................................................................................ OR577249 Dog 204 Hisar Haryana India.se
161 .........T...................................................................... OR577231 Dog 29 Meerut Uttar Pradesh In
161 .........T...................................................................... OR577243 Pup 190 Hisar Haryana India.se
```

```
    TATTATTAGGTGGTTATAGTGTAAGTGAACCAACTTTACAGAGGTTTTATATATTGCATTTTATACTTCCATTTGTATTA Majority
             250       260       270       280       290       300       310       320
241 ................................................................................ KC207825 USA.seq
241 ................................................................................ MK888703 Dog Futian China.seq
241 ................................................................................ MK888704 Dog Nanshan China.seq
241 ................................................................................ MK888705 Dog Baoan China.seq
241 ................................................................................ MK888706 Dog Longhua China.seq
241 ................................................................................ MK888707 Dog Longgang China.seq
241 ................................................................................ MK888708 Dog Longhua China.seq
241 ................................................................................ OR577229 Dog 27 Meerut Uttar Pradesh In
241 ................................................................................ OR577230 Dog 28 Meerut Uttar Pradesh In
241 ................................................................................ OR577232 Pup 68 Hisar Haryana India.seq
241 ................................................................................ OR577233 Pup 72 Ambala Haryana India.se
241 ................................................................................ OR577234 Pup 73 Ambala Haryana India.se
241 ................................................................................ OR577235 Pup 74 Ambala Haryana India.se
241 ................................................................................ OR577236 Pup 98 Hisar Haryana India.seq
241 ................................................................................ OR577237 Pup 101 Hisar Haryana India.se
241 ................................................................................ OR577238 Pup 108 Hisar Haryana India.se
241 ................................................................................ OR577239 Pup 109 Hisar Haryana India.se
241 ................................................................................ OR577240 Dog 120 Bareilly Uttar Pradesh
241 ................................................................................ OR577241 Dog 121 Bareilly Uttar Pradesh
241 ................................................................................ OR577242 Dog 122 Bareilly Uttar Pradesh
241 ................................................................................ OR577244 Pup 194 Hisar, Haryana India.s
241 ................................................................................ OR577245 Pup 199 New Delhi India.seq
241 ................................................................................ OR577246 Dog 200 New Delhi India.seq
241 ................................................................................ OR577247 Pup 201 Bareilly Uttar Pradesh
241 ................................................................................ OR577248 Pup 203 Hisar Haryana India.se
241 ................................................................................ OR577249 Dog 204 Hisar Haryana India.se
241 ................................................................................ OR577231 Dog 29 Meerut Uttar Pradesh In
241 ................................................................................ OR577243 Pup 190 Hisar Haryana India.se

    TTAGTTGTAGTTATTATTCATATATATTATTTACATAGATCTTCTAGTACAAATCCTTTATCTGGAATTGATTCTTGGTG Majority
             330       340       350       360       370       380       390       400
321 ................................................................................ KC207825 USA.seq
321 ................................................................................ MK888703 Dog Futian China.seq
321 ................................................................................ MK888704 Dog Nanshan China.seq
321 ................................................................................ MK888705 Dog Baoan China.seq
321 ................................................................................ MK888706 Dog Longhua China.seq
321 ................................................................................ MK888707 Dog Longgang China.seq
321 ................................................................................ MK888708 Dog Longhua China.seq
321 ................................................................................ OR577229 Dog 27 Meerut Uttar Pradesh In
321 ................................................................................ OR577230 Dog 28 Meerut Uttar Pradesh In
321 ................................................................................ OR577232 Pup 68 Hisar Haryana India.seq
321 ................................................................................ OR577233 Pup 72 Ambala Haryana India.se
321 ................................................................................ OR577234 Pup 73 Ambala Haryana India.se
321 ................................................................................ OR577235 Pup 74 Ambala Haryana India.se
321 ................................................................................ OR577236 Pup 98 Hisar Haryana India.seq
321 ................................................................................ OR577237 Pup 101 Hisar Haryana India.se
321 ................................................................................ OR577238 Pup 108 Hisar Haryana India.se
321 ................................................................................ OR577239 Pup 109 Hisar Haryana India.se
321 ................................................................................ OR577240 Dog 120 Bareilly Uttar Pradesh
321 ................................................................................ OR577241 Dog 121 Bareilly Uttar Pradesh
321 ................................................................................ OR577242 Dog 122 Bareilly Uttar Pradesh
321 ................................................................................ OR577244 Pup 194 Hisar, Haryana India.s
321 ................................................................................ OR577245 Pup 199 New Delhi India.seq
321 ................................................................................ OR577246 Dog 200 New Delhi India.seq
321 ................................................................................ OR577247 Pup 201 Bareilly Uttar Pradesh
321 ................................................................................ OR577248 Pup 203 Hisar Haryana India.se
321 ................................................................................ OR577249 Dog 204 Hisar Haryana India.se
321 ...A............................................................................ OR577231 Dog 29 Meerut Uttar Pradesh In
321 ...A............................................................................ OR577243 Pup 190 Hisar Haryana India.se

    TGTAACTAGATTTTATCCAGTTGTTATATTTAGTGATCTAAAGATGTTAACAACATTATTTATAGTTCTTGGTGTTCAAT Majority
             410       420       430       440       450       460       470       480
401 ................................................................................ KC207825 USA.seq
401 ................................................................................ MK888703 Dog Futian China.seq
401 ................................................................................ MK888704 Dog Nanshan China.seq
401 ................................................................................ MK888705 Dog Baoan China.seq
401 ................................................................................ MK888706 Dog Longhua China.seq
401 ................................................................................ MK888707 Dog Longgang China.seq
401 ................................................................................ MK888708 Dog Longhua China.seq
401 ................................................................................ OR577229 Dog 27 Meerut Uttar Pradesh In
401 ................................................................................ OR577230 Dog 28 Meerut Uttar Pradesh In
401 ................................................................................ OR577232 Pup 68 Hisar Haryana India.seq
401 ................................................................................ OR577233 Pup 72 Ambala Haryana India.se
401 ................................................................................ OR577234 Pup 73 Ambala Haryana India.se
401 ................................................................................ OR577235 Pup 74 Ambala Haryana India.se
401 ................................................................................ OR577236 Pup 98 Hisar Haryana India.seq
401 ................................................................................ OR577237 Pup 101 Hisar Haryana India.se
401 ................................................................................ OR577238 Pup 108 Hisar Haryana India.se
401 ................................................................................ OR577239 Pup 109 Hisar Haryana India.se
401 ................................................................................ OR577240 Dog 120 Bareilly Uttar Pradesh
401 ................................................................................ OR577241 Dog 121 Bareilly Uttar Pradesh
401 ................................................................................ OR577242 Dog 122 Bareilly Uttar Pradesh
401 ................................................................................ OR577244 Pup 194 Hisar, Haryana India.s
401 ................................................................................ OR577245 Pup 199 New Delhi India.seq
401 ................................................................................ OR577246 Dog 200 New Delhi India.seq
401 ................................................................................ OR577247 Pup 201 Bareilly Uttar Pradesh
401 ................................................................................ OR577248 Pup 203 Hisar Haryana India.se
401 ................................................................................ OR577249 Dog 204 Hisar Haryana India.se
401 .....................................A..........................A............... OR577231 Dog 29 Meerut Uttar Pradesh In
401 .....................................A..........................A............... OR577243 Pup 190 Hisar Haryana India.se
```

```
    TAACTTATGGTATTATACCTTTGTTTCAAGGTGATTGTGATAATTCTATTGCATCTAATCCACTTCAGACACCATTACAC Majority
             490       500       510       520       530       540       550       560
481 ................................................................................ KC207825 USA.seq
481 ................................................................................ MK888703 Dog Futian China.seq
481 ................................................................................ MK888704 Dog Nanshan China.seq
481 ................................................................................ MK888705 Dog Baoan China.seq
481 ................................................................................ MK888706 Dog Longhua China.seq
481 ................................................................................ MK888707 Dog Longgang China.seq
481 ................................................................................ MK888708 Dog Longhua China.seq
481 ................................................................................ OR577229 Dog 27 Meerut Uttar Pradesh In
481 ................................................................................ OR577230 Dog 28 Meerut Uttar Pradesh In
481 ................................................................................ OR577232 Pup 68 Hisar Haryana India.seq
481 ................................................................................ OR577233 Pup 72 Ambala Haryana India.se
481 ................................................................................ OR577234 Pup 73 Ambala Haryana India.se
481 ................................................................................ OR577235 Pup 74 Ambala Haryana India.se
481 ................................................................................ OR577236 Pup 98 Hisar Haryana India.seq
481 ................................................................................ OR577237 Pup 101 Hisar Haryana India.se
481 ................................................................................ OR577238 Pup 108 Hisar Haryana India.se
481 ................................................................................ OR577239 Pup 109 Hisar Haryana India.se
481 ................................................................................ OR577240 Dog 120 Bareilly Uttar Pradesh
481 ................................................................................ OR577241 Dog 121 Bareilly Uttar Pradesh
481 ................................................................................ OR577242 Dog 122 Bareilly Uttar Pradesh
481 ................................................................................ OR577244 Pup 194 Hisar, Haryana India.s
481 ................................................................................ OR577245 Pup 199 New Delhi India.seq
481 ................................................................................ OR577246 Dog 200 New Delhi India.seq
481 ................................................................................ OR577247 Pup 201 Bareilly Uttar Pradesh
481 ................................................................................ OR577248 Pup 203 Hisar Haryana India.se
481 ................................................................................ OR577249 Dog 204 Hisar Haryana India.se
481 .......C........................................................................ OR577231 Dog 29 Meerut Uttar Pradesh In
481 .......C........................................................................ OR577243 Pup 190 Hisar Haryana India.se

    ATAGTACCAGAATGGTATTTGCTTACATTTTATGCAACATTAAAATTATTTCCAAGTAAATTAGCAGGTTTAATTGCTAT Majority
             570       580       590       600       610       620       630       640
561 ................................................................................ KC207825 USA.seq
561 ................................................................................ MK888703 Dog Futian China.seq
561 ................................................................................ MK888704 Dog Nanshan China.seq
561 ................................................................................ MK888705 Dog Baoan China.seq
561 ................................................................................ MK888706 Dog Longhua China.seq
561 ................................................................................ MK888707 Dog Longgang China.seq
561 ................................................................................ MK888708 Dog Longhua China.seq
561 ................................................................................ OR577229 Dog 27 Meerut Uttar Pradesh In
561 ................................................................................ OR577230 Dog 28 Meerut Uttar Pradesh In
561 ................................................................................ OR577232 Pup 68 Hisar Haryana India.seq
561 ................................................................................ OR577233 Pup 72 Ambala Haryana India.se
561 ................................................................................ OR577234 Pup 73 Ambala Haryana India.se
561 ................................................................................ OR577235 Pup 74 Ambala Haryana India.se
561 ................................................................................ OR577236 Pup 98 Hisar Haryana India.seq
561 ................................................................................ OR577237 Pup 101 Hisar Haryana India.se
561 ................................................................................ OR577238 Pup 108 Hisar Haryana India.se
561 ................................................................................ OR577239 Pup 109 Hisar Haryana India.se
561 ................................................................................ OR577240 Dog 120 Bareilly Uttar Pradesh
561 ................................................................................ OR577241 Dog 121 Bareilly Uttar Pradesh
561 ................................................................................ OR577242 Dog 122 Bareilly Uttar Pradesh
561 ................................................................................ OR577244 Pup 194 Hisar, Haryana India.s
561 ................................................................................ OR577245 Pup 199 New Delhi India.seq
561 ................................................................................ OR577246 Dog 200 New Delhi India.seq
561 ................................................................................ OR577247 Pup 201 Bareilly Uttar Pradesh
561 ................................................................................ OR577248 Pup 203 Hisar Haryana India.se
561 ................................................................................ OR577249 Dog 204 Hisar Haryana India.se
561 ................................................................................ OR577231 Dog 29 Meerut Uttar Pradesh In
561 ................................................................................ OR577243 Pup 190 Hisar Haryana India.se

    GGGTGCTTTATTAGAATCATTGATACTTGTTGTCGAATCTAGAGCT                                   Majority
             650       660       670       680
641 ..............................................                                   KC207825 USA.seq
641 ..............................................                                   MK888703 Dog Futian China.seq
641 ..............................................                                   MK888704 Dog Nanshan China.seq
641 ..............................................                                   MK888705 Dog Baoan China.seq
641 ..............................................                                   MK888706 Dog Longhua China.seq
641 ..............................................                                   MK888707 Dog Longgang China.seq
641 ..............................................                                   MK888708 Dog Longhua China.seq
641 ..............................................                                   OR577229 Dog 27 Meerut Uttar Pradesh In
641 ..............................................                                   OR577230 Dog 28 Meerut Uttar Pradesh In
641 ..............................................                                   OR577232 Pup 68 Hisar Haryana India.seq
641 ..............................................                                   OR577233 Pup 72 Ambala Haryana India.se
641 ..............................................                                   OR577234 Pup 73 Ambala Haryana India.se
641 ..............................................                                   OR577235 Pup 74 Ambala Haryana India.se
641 ..............................................                                   OR577236 Pup 98 Hisar Haryana India.seq
641 ..............................................                                   OR577237 Pup 101 Hisar Haryana India.se
641 ..............................................                                   OR577238 Pup 108 Hisar Haryana India.se
641 ..............................................                                   OR577239 Pup 109 Hisar Haryana India.se
641 ..............................................                                   OR577240 Dog 120 Bareilly Uttar Pradesh
641 ..............................................                                   OR577241 Dog 121 Bareilly Uttar Pradesh
641 ..............................................                                   OR577242 Dog 122 Bareilly Uttar Pradesh
641 ..............................................                                   OR577244 Pup 194 Hisar, Haryana India.s
641 ..............................................                                   OR577245 Pup 199 New Delhi India.seq
641 ..............................................                                   OR577246 Dog 200 New Delhi India.seq
641 ..............................................                                   OR577247 Pup 201 Bareilly Uttar Pradesh
641 ..............................................                                   OR577248 Pup 203 Hisar Haryana India.se
641 ..............................................                                   OR577249 Dog 204 Hisar Haryana India.se
641 ..................G...........................                                   OR577231 Dog 29 Meerut Uttar Pradesh In
641 ..................G...........................                                   OR577243 Pup 190 Hisar Haryana India.se
```

Decoration 'Decoration #1': Hide (as '.') residues that match the Consensus exactly.

Decoration 'Decoration #2': Shade (with solid deep red) residues that differ from the Consensus.
