# Supplementary material for: Population genetics of Babesia vogeli based on the mitochondrial cytochrome b gene
Source: Sci Rep. 2024 Sep 20;14:21975. doi: 10.1038/s41598-024-72572-z (PMC11415385; doi:10.1038/s41598-024-72572-z)
Supplement: Supplementary file 3 — Supplementary Table 1. [file 41598_2024_72572_MOESM3_ESM.rtf]

Supplementary Table 1. Details of Babesia vogeli isolates infecting dogs of different countries used in the present study
S.N.	Place of sampling	Parasite	Country	Year of isolation	Host species	Isolate code/ Clone	Sequence length (bp)	Position	Accession No.	References	
1.		Meerut, Uttar Pradesh	Babesia vogeli	India	2023	Adult Dog	Dog 27/ -	693	8-693	OR577229	Current study	
2.		Meerut, Uttar Pradesh	Babesia vogeli	India	2023	Adult Dog	Dog 28/ -	693	8-693	OR577230 	Current study	
3.		Meerut, Uttar Pradesh	Babesia vogeli	India	2023	Adult Dog	Dog 29/ -	693	8-693	OR577231	Current study	
4.		Hisar, Haryana	Babesia vogeli	India	2023	Pup	Pup 68/ -	693	8-693	OR577232	Current study	
5.		Ambala, Haryana	Babesia vogeli	India	2023	Pup	Pup 72/ -	693	8-693	OR577233	Current study	
6.		Ambala, Haryana	Babesia vogeli	India	2023	Pup	Pup 73/ -	693	8-693	OR577234	Current study	
7.		Ambala, Haryana	Babesia vogeli	India	2023	Pup	Pup 74/ -	693	8-693	OR577235	Current study	
8.		Hisar, Haryana	Babesia vogeli	India	2023	Pup	Pup 98/ -	693	8-693	OR577236	Current study	
9.		Hisar, Haryana	Babesia vogeli	India	2023	Pup	Pup 101/ -	693	8-693	OR577237	Current study	
10.		Hisar, Haryana	Babesia vogeli	India	2023	Pup	Pup 108/ -	693	8-693	OR577238	Current study	
11.		Hisar, Haryana	Babesia vogeli	India	2023	Pup	Pup 109/ -	693	8-693	OR577239	Current study	
12.		Bareilly, Uttar Pradesh	Babesia vogeli	India	2023	Dog	Dog 120/ -	693	8-693	OR577240	Current study	
13.		Bareilly, Uttar Pradesh	Babesia vogeli	India	2023	Dog	Dog 121/ -	693	8-693	OR577241	Current study	
14.		Bareilly, Uttar Pradesh	Babesia vogeli	India	2023	Dog	Dog 122/ -	693	8-693	OR577242	Current study	
15.		Hisar, Haryana	Babesia vogeli	India	2023	Pup	Pup 190/ -	693	8-693	OR577243	Current study	
16.		Hisar, Haryana	Babesia vogeli	India	2023	Pup	Pup 194/ -	693	8-693	OR577244	Current study	
17.		New Delhi	Babesia vogeli	India	2023	Pup	Pup 199/ -	693	8-693	OR577245	Current study	
18.		New Delhi	Babesia vogeli	India	2023	Dog	Dog 200/ -	693	8-693	OR577246	Current study	
19.		Bareilly, Uttar Pradesh	Babesia vogeli	India	2023	Pup	Pup 201/ -	693	8-693	OR577247	Current study	
20.		Hisar, Haryana	Babesia vogeli	India	2023	Pup	Pup 203/ -	693	8-693	OR577248	Current study	
21.		Hisar, Haryana	Babesia vogeli	India	2023	Dog	Dog 204/ -	693	8-693	OR577249	Current study	
22.		Longhua	Babesia vogeli	China
	2019
	Dog	Longhua-17/ -	1075	206-891	MK888706
	Zhang, X.L., (Unpublished)
	
23.		Longgang	Babesia vogeli	China
	2019
	Dog	Longgang-18/ -	1075	206-891	MK888707	Zhang, X.L., (Unpublished)
	
24.		Longhua	Babesia vogeli	China
	2019
	Dog	Longhua-19/ -	1075	206-891	MK888708	Zhang, X.L., (Unpublished)
	
25.		Futian	Babesia vogeli	China
	2019
	Dog	Futian-1/ -	1092	206-891	MK888703	Zhang, X.L., (Unpublished)	
26.		Nanshan	Babesia vogeli	China
	2019
	Dog	Nanshan-11/ -	1092	206-891	MK888704	Zhang, X.L., (Unpublished)	
27.		Baoan	Babesia vogeli	China
	2019
	Dog	Baoan-14/ -	1092	206-891	MK888705	Zhang, X.L., (Unpublished)	
28.		-	Babesia vogeli	USA	2015	-	-/ -	5603	4451-5136	KC207825	Schreeg, M.E., Marr, H.S., Tarigo, J., Cohn, L.A., Bird, D.M., Scholl, E.H., Levy, M.G., Birkenheuer, A.J., (Unpublished)	
29.		-	Babesia canis	USA	2015	-	-/ -	5769	4586-5271	KC207822	Schreeg, M.E., Marr, H.S., Tarigo, J., Cohn, L.A., Bird, D.M., Scholl, E.H., Levy, M.G., Birkenheuer, A.J., (Unpublished)	
30.		Obihiro	Babesia ovata	Japan	2016	-	Miyake/ -	1092	202-887	LC146481	Yokoyama, N., (Unpublished)	
- denotes information not available.
