# Supplementary material for: Population genetics of Babesia vogeli based on the mitochondrial cytochrome b gene
Source: Sci Rep. 2024 Sep 20;14:21975. doi: 10.1038/s41598-024-72572-z (PMC11415385; doi:10.1038/s41598-024-72572-z)
Supplement: Supplementary file 4 — Supplementary Table 2. [file 41598_2024_72572_MOESM4_ESM.rtf]

Supplementary Table 2. Primers used to amplify specific fragments located between the 18S rRNA and the 28S rRNA genes of the large Babesia spp. by single-step PCR assays
Primer	Sequences	Parasite (Primers used)	Product size (bp)	
BAB1	5'-GTG AAC CTT ATC ACT TAA AGG-3'	- (Forward genus-specific)	-	
BAB3	5'-CTA CAC AGA GCA CAC AGC C-3'	B. canis (BAB1/BAB3)	746	
BAB4	5'-CAA CTC CTC CAC GCA ATC G-3'	B. vogeli (BAB1/ BAB4)	600	
BAB5	5'-AGG AGT TGC TTA CGC ACT CA-3'	B. rossi (BAB1/ BAB5)	342	
