# Supplementary material for: Population genetics of Babesia vogeli based on the mitochondrial cytochrome b gene
Source: Sci Rep. 2024 Sep 20;14:21975. doi: 10.1038/s41598-024-72572-z (PMC11415385; doi:10.1038/s41598-024-72572-z)
Supplement: Supplementary file 5 — Supplementary Table 3. [file 41598_2024_72572_MOESM5_ESM.rtf]

Supplementary Table 3. AMOVA analysis of population-genetic structure of B. vogeli populations based on the cyt b gene
Source of variation	Degree of freedom	Sum of squares	Variance components	Percentage of variation	Fixation Index (p value)	
Among populations	2	0.333	-0.06222 Va	-14.00	FST: -0.14000 (p= 1.00000)	
Within populations	25	12.667	0.50667 Vb	114.00		
Total	27	13.000	0.44444			
